# Transport in the brain studied with *in vivo* two-photon microscopy: the impact of spatial and temporal resolution

**DOI:** 10.1117/1.NPh.12.S2.S22806

**Published:** 2025-09-16

**Authors:** Nikolay P. Kutuzov, Martin Lauritzen

**Affiliations:** University of Copenhagen, Department of Neuroscience, Copenhagen, Denmark

**Keywords:** two-photon microscopy, brain, transport, diffusion, motion blur, resolution

## Abstract

Understanding molecular transport in the brain *in vivo* is essential for elucidating how the brain regulates its metabolism, how neurological pathologies develop, and why many brain-targeted drugs fail. Two-photon microscopy (TPM) is the gold standard for *in vivo* imaging in highly scattering tissues such as the brain. However, suboptimal use of TPM can compromise study outcomes due to the inherent challenges of *in vivo* imaging. We highlight the importance of optimizing both spatial and temporal resolution in TPM to ensure accurate data acquisition and interpretation. We compare TPM-based studies of molecular transport with traditional wide-field microscopy approaches, emphasizing how light scattering in brain tissue limits the effectiveness of the latter. We discuss the impact of motion blur—arising from diffusion of tracers or natural movement of cerebral vasculature—on image quality and offer practical strategies to mitigate these effects. In addition, we address the complexities of statistically analyzing noisy images, typically occurring due to low-photon budgets or the need for fast image recording in *in vivo* TPM. We conclude with a set of practical guidelines for effective data acquisition, aimed at facilitating the implementation of the concepts discussed. When properly optimized, TPM is a powerful tool capable of revealing fundamental mechanisms of brain transport and advancing our understanding of cerebral metabolism.

## 1 Introduction

### 1.1 Brain Metabolism and Transport

Brain metabolism and transport processes are closely interconnected, as the brain’s high energy demands rely on the efficient delivery of nutrients and removal of waste products. An average human brain contains on average 86 billion neurons,^1^ organized into a complex three-dimensional structure. Each of these cells requires nutrients and other essential molecules, e.g., oxygen and glucose, to be delivered and metabolic waste, e.g., carbon dioxide, lactate, various proteins, to be removed. The brain solves this logistics puzzle with three transport routes: (i) blood vessels, which efficiently transfer molecules over large distances to various regions in the brain; (ii) perivascular spaces of blood vessels, which help to remove brain waste^2^^,^^3^; and (iii) space between brain cells (extracellular space [ECS]), which distributes molecules between individual cells.^4^ If these transport routes break down, cells may starve or get poisoned by accumulating waste products. Thus, disruptions in either metabolic activity or transport processes can lead to neurological dysfunction, highlighting their critical interdependence in brain health and disease.

Despite comprising only 2% of our body mass, the human brain consumes ∼20% of our metabolic energy.^5^ This makes it one of the most energy-intensive organs in the body. Unlike most other tissues, which can extract and consume a variety of nutrients from the blood, the normal brain is almost exclusively dependent on glucose for energy metabolism. The rapid turnover of brain ATP is fueled by glucose oxidation. In the resting human brain, glucose oxidation produces 300 to 400 mL of water per day. About 100 mL of this is secreted and constitutes an important part of the brain’s interstitial fluid flow.^6^ This flow distributes nutrients, metabolites, and peptides among nerve cells and clears brain waste. Interestingly, the secretion rate of metabolic water matches the lymphatic flow,^7^^,^^8^ which provides independent support for the concept of metabolic water as a brainwashing mechanism.^6^ In simple terms, brain glucose oxidation not only fuels information processing, computation, and coding but also fluid production. Metabolic water, which is produced continuously, seeps out and coats the brain’s surface.

In the resting state, most metabolic water is produced by mitochondria in neurons. However, during increases in brain activity, water output decreases because neurons begin consuming water during energy turnover. The turnover of one molecule of ATP requires the uptake of one molecule of water. Remarkably, astrocytes (support cells) have significant water-related functions due to their substantial glycogen stores and energy bursts.^6^ Thus far, there have been no detailed studies of metabolic water production and secretion in the central nervous system. In particular, the water kinetics (i.e., the location of permeation of metabolic water across neuronal cell membranes) and water dynamics (i.e., the transport of metabolic water in the brain’s interstitial space) are incompletely understood. Furthermore, only limited *in vivo* data exists on mechanisms of transport in the brain interstitial spaces,^4^^,^^9^^,^^10^ in the brain perivascular spaces,^2^^,^^3^^,^^11^ and across the blood-brain barrier.^12^^–^^14^ However, to prepare for these complex tasks, we must optimize our imaging techniques to the limit of their performance.

### 1.2 Methods to Study Transport in the Brain

A variety of techniques have been employed to investigate molecular transport in the brain *in vivo*, including, but not limited to, real-time iontophoresis (RTI),^3^^,^^15^ integrative optical imaging (IOI),^9^^,^^10^ fluorescence recovery after photobleaching (FRAP),^16^ fiber photometry,^17^^,^^18^ and two-photon microscopy (TPM).^3^^,^^11^^–^^14^^,^^19^ Among these, RTI is unique in that it does not rely on optical measurements and is capable of quantifying the volume fraction of the brain’s ECS. However, RTI has a limited spatial resolution of ∼100  μm and is restricted to small ions for which ion-selective microelectrodes can be made.

By contrast, optical methods such as fluorescence microscopy enable studying a wide range of fluorescent molecules across both large (∼100  μm) and small (<10  μm) spatial scales in the brain.^4^ High spatial resolution is particularly important for probing local transport phenomena, such as those occurring in the vicinity of individual cells or blood vessels. For instance, super-resolution imaging of brain slices has revealed that the local geometry and physical properties of the ECS, such as viscosity, can vary significantly on the micrometer scale.^20^^,^^21^ Studying local transport *in vivo* in the brain presents at least two challenges: (i) the brain’s turbid nature degrades spatial resolution and (ii) the intrinsic motion of tracer molecules and blood vessels can introduce significant motion blur, compromising image quality. In the following sections, we address both of these challenges in detail.

## 2 Spatial Resolution

Spatial resolution in optical microscopy refers to the ability of a microscope’s objective to distinguish two closely spaced objects. In microscopic images recorded with low spatial resolution, the fine structure of the sample is hidden as the fine details are blurred. Higher spatial resolution means a higher level of detail, i.e., finer structural features are observed within the sample. The spatial resolution of a microscope is influenced by the wavelength of light used, the numerical aperture of the objective lens (NA), the immersion medium, and, last but not least, the sample’s optical properties. Understanding and optimizing spatial resolution is crucial to recording high-quality data and increasing the interpretability of the results.

### 2.1 Point-spread Function

Spatial resolution in microscopy is directly linked to the shape and size of the point-spread function (PSF), which is the output you get when imaging an object smaller than the wavelength of light you illuminate the object with. PSF can be estimated theoretically or measured experimentally, for example, by imaging a 40-nm fluorescent bead, or you can even see the PSF by eye if you focus laser light inside a solution of a fluorescent dye as shown in Fig. 2b in Ref. 22. Fluorescence intensity is highest at the objective’s focus and decreases gradually as we move away from it. PSF mathematically describes this excitation volume, within which fluorescence is generated. PSF relates to the spatial resolution of a microscope: lateral and axial resolutions of a microscope are commonly defined as the PSF’s full width at half maximum (FWHM) in these directions.

Thus, the PSF serves as a descriptor of a microscope’s spatial resolution, defining how sharp or blurred the image will be. It plays a central role in describing the image formation and is widely used for image analysis, e.g., in deconvolution microscopy and super-resolution microscopy.

**Fig. 1:**
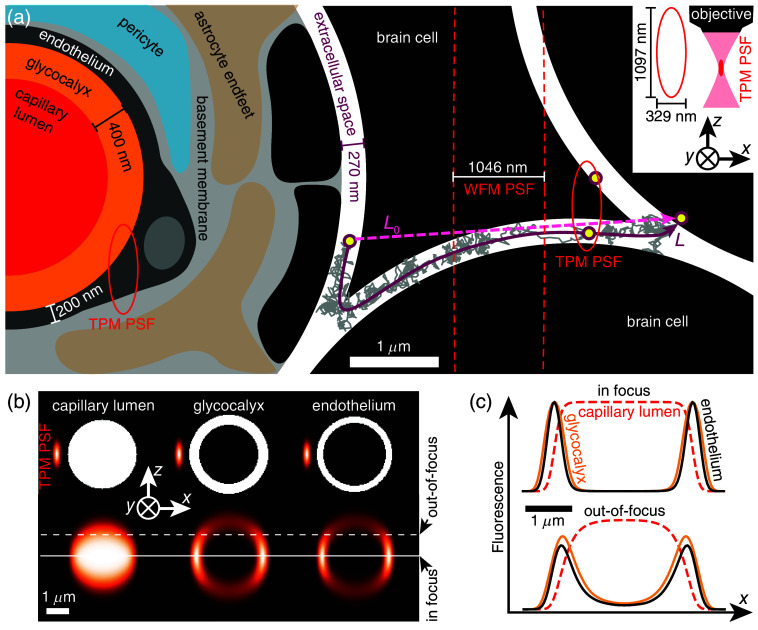
(a) Spatial resolution visualized with point-spread functions (PSF) for wide-field microscopy (WFM PSF) and two-photon microscopy (TPM PSF). PSFs are shown as ellipses with their smallest and largest diameters equal to FWHM of the PSF in the focal plane (xy–plane, ${\mathrm{FWHM}}_{xy}$) and along the z–axis (see inset, ${\mathrm{FWHM}}_{z}$), respectively. For WFM (dashed red ellipse) ${\mathrm{FWHM}}_{xy}=1046\;\mathrm{nm}$ and ${\mathrm{FWHM}}_{z}=16\;\mu m$, as estimated using (Ref. 9, Eqs. 4–5 and Fig. 2) with 610-nm excitation and NA=0.3 (water immersion). For TPM (solid red ellipse) ${\mathrm{FWHM}}_{xy}=329\;\mathrm{nm}$ and ${\mathrm{FWHM}}_{z}=1097\;\mathrm{nm}$, as estimated using (Ref. 22, Fig. 4c) with 900-nm excitation and NA=1.05 (water immersion). We show a 200-nm-thick vascular endothelium (with a locally bulging nucleus), based on the measurements with electron microscopy, which range roughly from 50 to 300 nm;^23^^,^^24^ 400-nm-thick glycocalyx, based on *in vivo* measurements in capillaries;^25^^,^^26^ and 270-nm-wide ECS, based on the median width of ECS observed in brain slices.^21^ Gray zig-zag curve: an example of a trajectory of a molecule forced to travel a longer distance (L) compared with the shortest distance $L_{0}$ inside an ECS channel, connecting two brain cells (dashed pink). This elongation of the traveling path contributes to slower transport of molecules in the brain, quantified by the tortuosity, $\lambda =\sqrt{\frac{D}{D^{*}}}$, where D and $D^{*}$ are diffusion coefficients of a molecule in water (gel) and in the brain, respectively.^4^^,^^9^ (b) Three examples of image formation in TPM. The top row shows binary masks simulating the distribution of fluorescent molecules in a capillary lumen (left), glycocalyx (middle), and endothelium (right). The sizes of these structures are the same as in panel (a). Bottom row shows the expected images of the three structures, obtained by convolving the binary masks with the TPM PSF. (c) Line profiles of fluorescence extracted along the white solid line (capillary in focus) and along the white dashed line (capillary out-of-focus) in the simulated images in panel (b). Note how the width of the glycocalyx profile is only slightly wider compared with the endothelium profile despite a factor of two difference in width [top row in panel (b)].

When planning an experiment, it is essential to compare the spatial resolution of the microscope with the typical sizes of structures in the sample. This comparison helps determine in advance which structures will be visible in the recorded images and how they are likely to appear. Figure 1(a) schematically illustrates various cellular and extracellular structures, along with their typical dimensions, in a region of the brain adjacent to a capillary. By placing the PSF alongside these structures, it becomes evident that neither wide-field microscopy (WFM) nor TPM can resolve individual channels of the brain’s ECS using single-particle tracking or bulk dye loading. This limitation arises because the TPM PSF extends over approximately a micrometer along the z-axis, allowing it to collect fluorescence from two distinct fluorescent molecules (represented as yellow circles within the red ellipse) located in separate ECS channels. These vertically stacked channels become indistinguishable in the recorded image, appearing as a single, blurred structure.

The PSF is valuable not only for defining the optical resolution of an imaging system but also for predicting how specific geometrical structures will appear in recorded images. This prediction can be achieved with just a few lines of code by *convolving* the PSF with the spatial distribution of fluorescent molecules. For example, Fig. 1(b) shows the expected distribution of fluorescence across a vessel’s cross-section by convolving the Gaussian TPM PSF^22^^,^^27^^,^^28^ with a uniform distribution of a dye on a circle. This becomes useful when taking a line-scan across a capillary, filled with a fluorescent dye, for measuring its diameter: a capillary in focus images as a line distribution of fluorescence with a flat top [Fig. 1(c)]. If the line distribution is more peaky, without a flat central region, this is likely because the capillary moved away from the focus.

Similarly, one can model the expected fluorescence distribution of a ring to simulate images of a vessel cross-section with uniformly labeled glycocalyx or vascular endothelium. Notably, the simulated images of a capillary with labeled glycocalyx and endothelium appear quite similar [Fig. 1(b)], and their corresponding line profiles [Fig. 1(c)] show comparable widths—even though the glycocalyx is twice as thick. This highlights how the PSF influences the apparent shape and size of structures in experimental images: it is not possible to accurately estimate the width of the glycocalyx or endothelium without accounting for the blurring introduced by the PSF. Up to this point, we have considered only the theoretical PSF, which depends solely on the objective lens and excitation wavelength. In the following section, we demonstrate how the sample itself (e.g., brain tissue) can significantly alter the PSF.

### 2.2 Spatial Resolution and Point-Spread Function in Wide-Field Microscopy

#### 2.2.1 Brief theory of integrative optical imaging

**Fig. 2:**
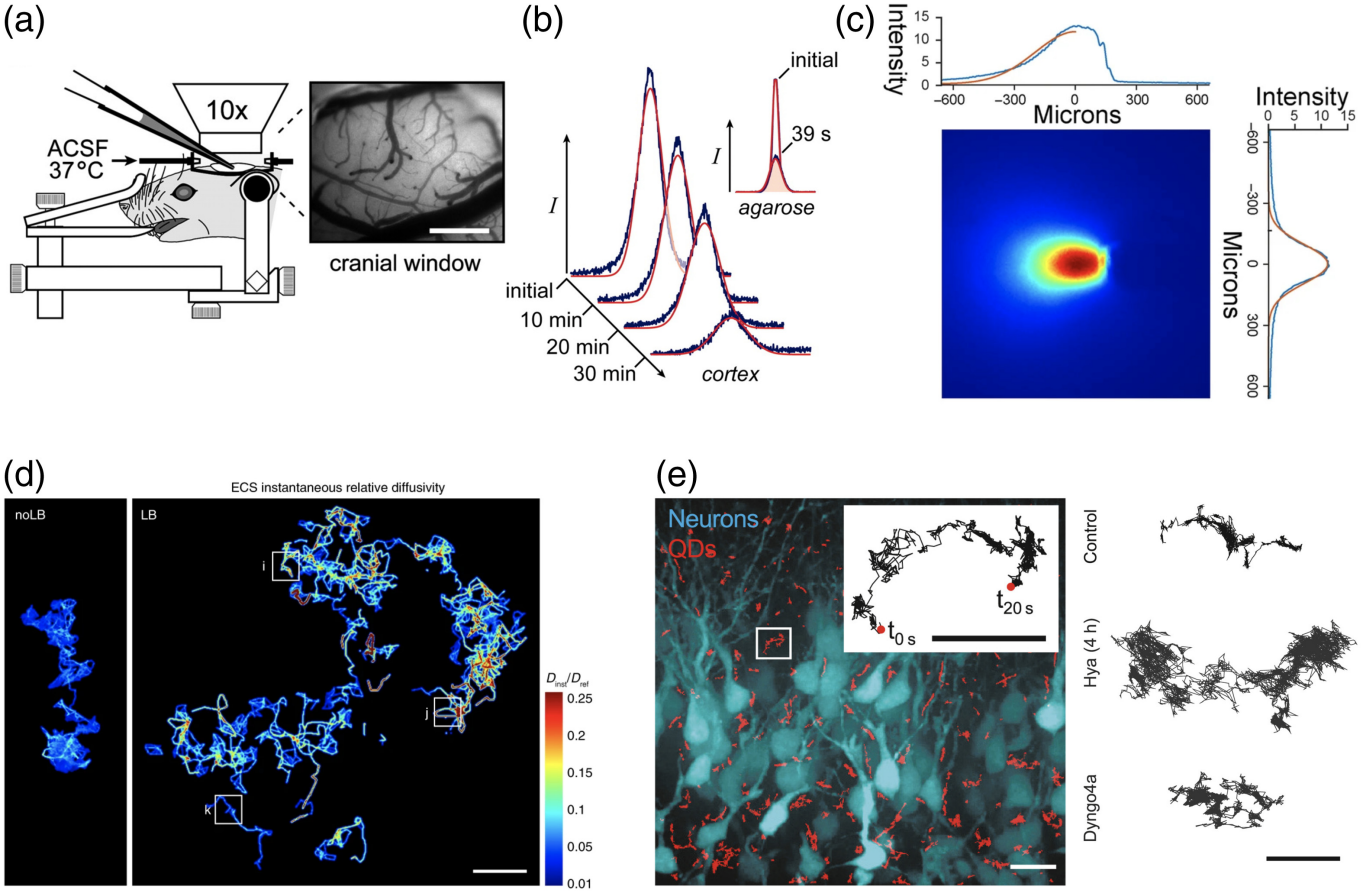
Transport in the brain studied with integrative optical imaging (IOI), fiber photometry, and single-particle tracking. (a) In IOI imaging, fluorescence dye is injected in the brain parenchyma (inset shows the craniotomy) of an anesthetized rat.^10^ Scale bar: 500  μm. (b) In IOI, diffusion coefficients of quantum dots (QDs) in the brain are estimated by fitting Gaussians (red curves) to the line-intensity profiles (extracted from each image) taken across the diffusing cloud of QDs (blue). The inset shows line-intensity profiles of diffusing QDs recorded in an agarose gel, where after only 39 s, the peak fluorescence intensity drops by >50%. In the brain, such a decrease takes more than 20 min—the transport of molecules in the brain is markedly slowed down, compared with agarose. Note the fat tails in the experimental line profiles, which cause the deviation from the fitted Gaussians.^10^ (c) Distribution of fluorescence intensity (red—high, blue—low) inside a brain slice, seen from an objective above, where the excitation light is propagating from the tip of a 200-μm-diameter optical fiber, located at y≈0  μm and x≈150  μm.^17^ Top and right insets show 1D line profiles across the fluorescent blob. Note the fat tails of the fluorescence intensity distributions that are not described by the fitted Gaussians. (d) Single trajectories of single-walled carbon nanotubes (CNs) in brain slice ECS of a Parkinsonian (LB) and a healthy control mouse (noLB) showing how relative diffusion coefficients of CNs increase in LB mice. Scale bar is 2  μm. (e) Single trajectories of QDs in brain slice ECS (left; scale bar: 15  μm) and examples of how treatments of ECS (right; scale bar: 5  μm) with hyaluronidase (Hya), which digests extracellular matrix, and Dyngo-4a, which blocks endocytosis of QDs, affect diffusion of QDs. The inset in the left image shows a single QD trajectory. Scale bar: 5  μm. Panels (a) and (b) are from (Ref. 10, Figs. 1B and 4B), PNAS. Panels (a) and (b) are Copyright (2006) National Academy of Sciences. Panel (c) is from (Ref. 17, extended data Fig. 4b), Nat. Neurosci. Panel (d) is from (Ref. 29, Fig. 2cf), Nat. Commun. Panel (e) is from (Ref. 30, Fig. 1AB), Cell Rep. Panels c–e are reprinted with permission.^31^

IOI is a subtype of WFM that is used to estimate diffusion coefficients of fluorescently labeled molecules in the brain ECS *in vivo*.^9^ The method [Figs. 2(a)–2(c)] is based on the following assumptions:

1. The fluorescent molecules diffuse in the brain ECS with no cell uptake or any other type of efflux from the ECS (i.e., clearance), no photobleaching or any other chemical reaction, i.e., the molecules are conserved as they diffuse.
2. The brain is homogeneous, isotropic, and does not change in time, as experienced by the diffusing molecules. If this is the case, the diffusion is characterized by a single parameter—effective diffusion coefficient, $D^{*}$, which is related to the diffusion coefficients in the free space (water or agarose gel), D, through the tortuosity, $\lambda =\sqrt{\frac{D}{D^{*}}}$ [Fig. 1(a)]. With the first assumption, this means that the concentration of fluorescent molecules follows the diffusion equation (Fick’s second law).
3. The injection volume is so small that the concentration of molecules at the start of the measurement is a Gaussian, as if at an earlier time, all molecules were concentrated in a single point in space.

From these assumptions, it follows that the concentrations of the fluorescent molecules are a rotationally symmetric 3D Gaussian distribution centered on the point of injection at all times. The recorded image is a 2D distribution of fluorescence intensity obtained by convolving the 3D distribution of the fluorophores (concentration in xyz) with the PSF of the microscope [Fig. 1(a)].

Note that IOI is simply a type of WFM that uses a low-NA objective: an objective with NA = 0.3 results in a PSF that has FWHM along the z-axis of 16  μm. Nicholson and Tao argue that this approximately amounts to integrating (“summing”) the concentration of fluorophores over the principal axis (z-axis in Fig. 4) of the objective (Fig. 4 in Nicholson and Tao^9^). Because integration of a 3D Gaussian over z-axis yields a rotationally symmetric (about the origin, i.e., point of injection of the dye) 2D Gaussian, its values are the same along any straight line going through the origin. Estimation of the diffusion coefficients is then done by fitting a 1D Gaussian to line profiles of intensity [Fig. 2(c)] and then fitting a first-degree polynomial to half the variance of the fitted Gaussian, $\frac{1}{2}\mathrm{Var}(t)$, as a function of time, t. The slope of the fitted line gives the diffusion coefficient D (Figs. 7 and 9 in Nicholson and Tao^9^).

#### 2.2.2 Theory meets data: How does the brain tissue affect the PSF?

Figure 2(b) shows deviations of the “skinny” tails of the fitted Gaussians from the “fat” tails of the experimental line profiles of fluorescence. Similar fat tails are seen in other publications employing IOI.^9^^,^^34^^–^^37^ Importantly, as the intensity of the central peak decreases, the tails have more influence on the fit, which can lead to the deviation of the 1/2 Var(t) curve from a first degree polynomial, e.g., as seen in Figs. 2DC and 3 in Xiao et al.^37^ The authors explained the tails as due to anomalous diffusion.^37^

Another explanation for the fat tails is that they are the property of the initial non-Gaussian distribution of the dye after its injection in the sample. This explanation is supported by the presence of the tails on the initial profiles of fluorescence, such as the one in Fig. 2(b). If this explanation is correct, then optimal estimation of the diffusion coefficients should be done by changing the initial condition from a Gaussian to a fat-tailed distribution. This distribution is then propagated in time by convolution with a Gaussian with a variance defined by the diffusion coefficient and the time passed from the injection.

It has also been suggested that the fat tails arise due to light scattering in the brain tissue.^34^^–^^36^ Microscopic imaging of fluorescent molecules in biological tissues has several important features that complicate the interpretation of the recorded data in comparison with imaging in an agarose gel [inset in Fig. 2(b)]. Consider a piece of brain tissue, whose structure can be roughly approximated by a collection of various-sized microscopic objects. If the thickness of a sample exceeds a mean-free path for an incident light, then it can be scattered (once or multiple times), which affects its propagation direction. Consider a molecule that absorbs a photon focused by a microscope’s objective and emits a fluorescence photon. If it travels along a straight line (without scattering) from the sample into the collecting objective, its projection onto the CCD camera (wide-field imaging) will reflect its actual position inside the sample. Such photons are called ballistic, and they form a diffraction-limited image. However, if the emitted photon is collected by an objective after it scatters, changes direction, its location on the image may not reflect the actual position of the molecule that emitted the photon.^38^^,^^39^ Thus, collecting scattered photons leads to an additional blurring of an image.

To minimize the effect of the scattering, some authors fit the Gaussian only to the central part of the experimental line distributions, discarding their tails,^35^^,^^36^ e.g., in Fig. 2C of Thorne et al.^35^ This approach is motivated by the idea that a sharp peak in the middle of the line distributions reflects an actual concentration of the dye close to the injection point and is due to ballistic photons. Fat tails can be due to the multiple scattered photons and thus do not show the actual concentration distribution of the dye. The disadvantage of separating ballistic photons from scattered ones based on an intensity threshold is that the particular choice of the cutoff threshold for the tails is arbitrary, and choosing a different threshold will affect the results. Another disadvantage is that cutting off the tails, caused by light scattering, does not remove the contribution of the light scattering to the central part of the peak, which should be accounted for.

Supporting the scattering origin of the fat-tailed fluorescence distributions, Fig. 2(c) shows the distribution of fluorescence intensity (visualized by an objective from the top), excited by an optical fiber inserted in a brain slice.^17^ Notice the fat tails of fluorescence (blue) in the vertical image direction perpendicular to the 200-μm-diameter fiber (oriented horizontally) and compare them with the fitted Gaussian (orange). Importantly, these tails are not due to a fat-tailed distribution of the excitation light propagating from the fiber. Stujenske et al.^40^ modeled and compared propagation of light in transparent media and in the brain and showed a drastic effect of scattering on the light distribution (Ref. 40, Fig.1C), which can explain fat-tailed distributions of fluorescence observed in the IOI method^9^ and in fiber photometry.^17^

The example above illustrates how the spatial resolution of a microscope depends on both the microscope and the sample—the brain. Understanding the optical properties of the brain (scattering, absorption) is crucial for optimal recording and interpretation of the experimental data. Despite the challenge of light scattering, the IOI method is very useful to study and quantify transport in the brain *in vivo* on the spatial scale of hundreds of micrometers, limited by the width of the initial (after the injection) distribution of the diffusing dye. To study how molecules diffuse on the microscale, near single brain cells, a variety of single-particle tracking methods have been developed.

### 2.3 Tracking Single Nanoparticles with Wide-field Microscopy

Tracking single molecules belongs to a group of imaging techniques—super-localization microscopy—that surpasses the diffraction limit of conventional light microscopy, reaching nanometer resolution. It achieves this by locating centers of individual fluorescent molecules. These techniques rely on imaging only a sparse subset of fluorophores at a time, localizing them with nanoscopic precision, and, finally, reconstructing a super-resolved image from the estimated single locations.

Single-walled carbon nanotubes (CNs) have been used to study diffusion in ECS at the nanoscale in slices of the brains of mice and rats.^20^^,^^29^^,^^41^ Soria et al.^29^ have demonstrated increased diffusivity and width of ECS in a mouse model of synucleinopathy [Fig. 2(d)], linking changes in the brain ECS with neuropathology. As an alternative to CNs, single quantum dots (QDs) can be tracked in brain slices.^30^ QDs are smaller than CNs and their shape resembles natural molecules (proteins) better than CNs, which makes QDs more relevant for some applications, e.g., for drug delivery. Grassi et al.^30^ demonstrated heterogeneity of the brain ECS in different regions of the hippocampus and showed how QD diffusion was affected by shedding of the extracellular matrix [Fig. 2(e)].

Super localization of nanoparticles established itself as a very powerful method, allowing for to study of transport at the nanoscale in health and in pathology. The quality of the produced results, however, can be further improved with state-of-the-art localization analysis. The majority of the used localization algorithms are based on standard approaches based on custom custom-written algorithms, or software packages used for super-localization microscopy. Most of the methods are based on fitting 2D Gaussian functions to distributions of fluorescence on recorded images.^20^^,^^29^^,^^30^^,^^41^ Although these methods are considered standard and most commonly used, they can deliver suboptimal super-localization precision. Well-developed protocols exist for super-localization of fluorescent beads and molecules in images acquired with a wide-field microscope equipped with a CCD or EMCCD camera.^42^^,^^43^ These localization routines use all of the information in the recorded images, yielding optimal super-localization, based on the correct physical description of the image formation and statistical description of the process of photon detection, multiplication, and the generation of noise on the images.^42^

To ensure the highest quality results, super-localization should be followed by the optimal analysis of single nanoparticle trajectories. Mean-squared displacement (MSD) analysis for the estimation of diffusion coefficients is the standard, widely used approach.^44^^,^^45^ It is, however, challenging to apply it for the analysis of single trajectories because of correlations of the MSD values.^45^^,^^46^ Tracking small nanoparticles, e.g., QDs compared with CNs, in the brain ECS is further challenging as they quickly move out of focus, interrupting recorded trajectories. Covariance-based estimator (CVE) is an explicit and unbiased estimator, and it is especially useful for the analysis of short trajectories.^46^ Importantly, the choice of the estimator for the diffusion coefficient also depends on the type of motion studied, e.g., free diffusion, diffusion on a moving substrate,^46^ or non-Gaussian diffusion.^47^

Single nanoparticle tracking has quickly become a standard tool to study transport at the level of single brain cells and achieve nanoscale resolution. Doing super-localization on images recorded with WFM, however, limits these methods to brain slices only. *In vivo* imaging in the brain of living mice requires a method that can deliver 3D resolution in scattering tissue. TPM is one of these methods.

### 2.4 Spatial Resolution and Point-Spread Function in Two-Photon Microscopy

A standard two-photon microscope is a scanning microscope: it images a specimen by scanning it with a focused laser beam, point by point, and collecting all incoming fluorescence photons [Fig. 4(a)]. Under ideal imaging conditions (no aberrations), a microscope’s PSF has two symmetries: (i) rotational symmetry around the optical axis of the objective (z-axis) and (ii) symmetry about xy-plane. Assuming no aberrations and matching refractive indexes (RI) of the specimen and the immersion medium, analytical expressions for 3D PSF for TPM have been derived: see, for example, Eqs. (1)–(3) in Ref. 27. Gaussian approximation of TPM PSF has been tested experimentally and is most widely used.^12^^,^^28^^,^^48^

When imaging conditions are not perfect, such as when imaging in the brain, PSF symmetries are broken. If RI of the specimen differs from RI of the immersion medium or if the objective is not properly corrected for the coverslip, PSF loses symmetry about xy-plane and its maximum flattens as PSF widens in xy-plane and especially along z-axis.^49^^–^^51^ To understand how a RI-mismatch affects PSF, one can use theoretical models^50^^,^^52^^,^^53^ or (and) experimental data. Using a common 1.5-thickness coverslip distorts excitation volume measured with a high-NA objective (Ref. 51, Fig. 2b). Here, we used “excitation volume” instead of “PSF” because Ji et al. collected images of 0.2-μm beads in water, and these beads are not small enough to be considered a point source of light. But, of course, it is totally fine to study distortions of PSF by visualizing large beads because they produce images with higher contrast, as long as the distribution of fluorescent dye inside the bead is spherically symmetric about the bead’s center. Although PSF symmetry about xy-plane can be broken by RI mismatch, PSF rotational symmetry about z-axis is conserved if the RI of the medium is uniform throughout its volume.

**Fig. 3:**
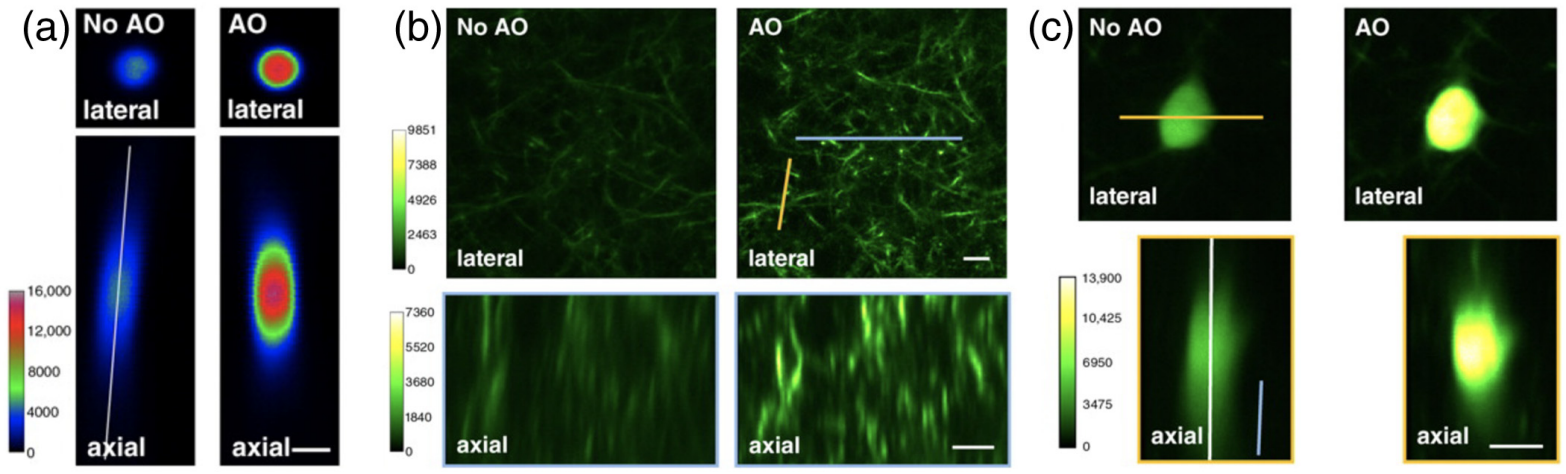
Brain-induced optical aberrations and their correction with adaptive optics (AO). (a) Images of a 2-μm-diameter bead in XY-plane (lateral, top) and in XZ-plane (axial, bottom) 170  μm below the brain surface without and with AO. (b) GFP-expressing dendritic processes in XY-plane (lateral, top) and in XZ-plane (axial, bottom) without and with AO. (c) GFP-expressing neurons in XY-plane (lateral, top) and in XZ-plane (axial, bottom) without and with AO, 110  μm below the brain’s surface. Brain-induced aberrations may result in, e.g., distortion of imaged objects (a), lower spatial resolution (bc), and lower image contrast (bc). Scale bars: 2 and 10  μm for (a) and (bc), respectively. All panels are from (Ref. 54, Fig. 1bdg), PNAS, with permission.^31^

In the brain—a scattering medium with nonuniform RI, both symmetries of the PSF can be broken (Fig. 3). Cell membranes, cell bodies and nuclei, blood vessels, and extracellular structures all contribute to nonuniform RI of the brain and make it prohibitively difficult to theoretically model it. Nevertheless, adaptive optics has been successfully used to correct brain-induced aberrations.^51^^,^^54^^–^^56^ Figure 3(a) shows an image of a 2-μm bead inside the brain before and after correction: brain tissue can distort the PSF, resulting in images with low contrast. TPM with adaptive optics increases the resolution of a TPM and enhances contrast of recorded images, which is especially relevant when imaging deep layers of the brain (Fig. 3).^54^^–^^56^ For a more detailed description of state-of-the-art optical tools for multiphoton brain imaging, we refer the reader to comprehensive reviews.^57^^,^^58^

Degradation of PSF when imaging in the brain puts limits on applications that demand high image quality, for example, super-resolution imaging.^56^ For example, TPM PSF can lose rotational symmetry around z-axis [Fig. 3(a)]. When localizing single particles or molecules, such an oblique PSF can bias estimated locations: the centroid of the measured intensity distribution depends on the z-coordinate of the molecule relative to the focus of the objective. Therefore, ideally, PSF should be measured inside the brain at regions where measurements are taken. Similar to WFM, the brain itself decreases the spatial resolution of TPM, especially at deep layers of the cortex. Even within several hundred micrometers under the brain surface, the spatial resolution can decrease 1.8 and 3 times in the lateral and axial dimensions, respectively, as measured with QDs in the brain.^12^ When characterizing PSF *in situ*, e.g., near a blood vessel in the brain, the QD is assumed to be a point source of light that does not move. These assumptions are challenged in *in vivo* imaging: QDs can aggregate and lump together so that they cannot be regarded as point sources of light, and QDs move with the brain, which further blurs their images. The result will be an overestimated size of the PSF, or equivalently, an underestimated spatial resolution. Nevertheless, having a theoretical estimate of the spatial resolution, supplied with experimentally measured spatial resolution in the brain at a desired depth, can help experimental planning and interpretation of the obtained results.

## 3 Temporal Resolution

### 3.1 Images Recorded with TPM are not Images but Time Series

**Fig. 4:**
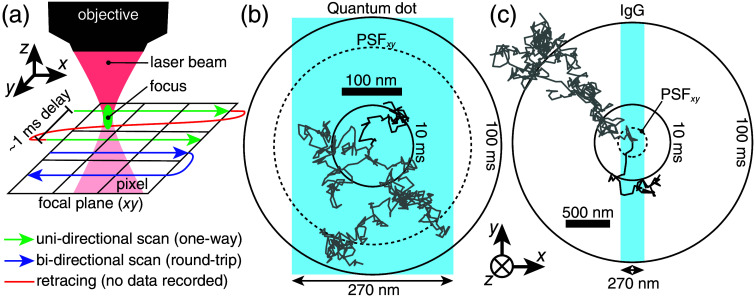
Temporal scales of diffusion in the brain compared with the scanning speed of TPM. (a) TPM records images line-by-line (green lines) by moving the laser beam, focused by the objective (red), relative to the imaged sample. (b)–(c) Simulated 10-ms (black zigzag curve) and 100-ms (gray zigzag curve) random-walk trajectories in free space of a 35-nm diameter QD^10^ (b), and 150-kDa (≈11  nm^32^) IgG (c) relative to the median width of brain ECS^21^ (blue rectangle) and to the microscope’s PSF, shown with dashed circle with diameter equal to the FWHM of the PSF, ${\mathrm{PSF}}_{xy}=329\;\mathrm{nm}$ [see Fig. 1(a)]. For the simulations, diffusion coefficients, D, for the QD and IgG were D=0.167^10^ and $D=6.7\;\mu m^{2}/s$.^33^ Black solid circles, marked 10 and 100 ms, show the area containing 50% of random walkers at times t=10  ms and t=100  ms, which all started diffusing at t=0 at the center of the circles.

Image acquisition in WFM and TPM is principally different: WFM records all image pixels simultaneously, whereas TPM records images by scanning the sample with a laser beam pixel-by-pixel as shown in Fig. 4(a). This results in a time delay between recordings of different pixels. For a unidirectional galvo-scanning (standard scanning option in TPM), the pixel exposure time, $t_{\mathrm{pix}}∼2\;\mu s$ ($t_{\mathrm{pix}}$ is also the time delay between recordings of two consecutive pixels). The time delay between recording the last pixel of one line to the first pixel of the following line, called retracing time, $t_{\mathrm{rtr}}∼1\;\mathrm{ms}$. In this case, an image is a time series taken with two different time steps: one for pixels within a single image row, another for pixels on different image rows. This may sound similar to a technical detail, but thinking of TPM images as time series helps better interpret recorded images and recognize when the images are expected to be distorted by the motion blur.

### 3.2 Motion Blur Due to Diffusion

Consider tracking a single QD and a single IgG molecule in the brain ECS with TPM [Figs. 4(b) and 4(c)]. Say we take a square image 1×1  μm, 20×20  pixels (50-nm wide pixels). With unidirectional scanning [Fig. 4(a)], one line is recorded within $t_{\text{line}}=t_{\mathrm{pix}}\cdot n_{x}+t_{\mathrm{rtr}}=2\;\mu s/\text{pixel}\cdot 20\;\text{pixels}+1\;\mathrm{ms}=1.04\;\mathrm{ms}$. Both the QD and the IgG molecules are much smaller than the wavelength of the excitation light, λ=900  nm, so they should image as a diffraction-limited (for simplicity, assuming that the brain does not distort the PSF) Gaussian-like blob with FWHM of 329 nm [Fig. 1(a)]. This blob spans 329  nm/50  nm≈7  pixels both in x and y directions. To record an image of the blob, we need to take at least seven-line-tall image, which will take $7\cdot t_{\text{line}}=7.3\;\mathrm{ms}$. For free diffusion, the root mean-squared displacement of a molecule, $\mathrm{RMSD}=\sqrt{2Dt}$ depends on the molecule’s diffusion coefficient, D, and the elapsed time, t. For a QD ($D=0.167\;\mu m^{2}/s$^10^) and an IgG ($D=6.7\;\mu m^{2}/s$^33^), RMSD (t=7.3  ms) will be ${\left(2\times 0.0073\;s\times 0.167\;\mu m^{2}/s\right)}^{1/2}=49\;\mathrm{nm}$ and ${\left(2\times 0.0073\;s\times 6.7\;\mu m^{2}/s\right)}^{1/2}=313\;\mathrm{nm}$, respectively.

The results are drastically different for a QD and for an IgG molecule. While we are imaging a QD, it will move roughly by one pixel (pixel width = 50 nm), which is a 49 nm/329 nm = 15% displacement relative to the diffraction-limited blob size. As a result, the distribution of fluorescence of the QD will still resemble a blob, but it will be wider than expected for a stationary light source (Ref. 59, Fig. 2B). By contrast, an IgG molecule will move 313  nm/329  nm=95% of its diffraction-limited blob size. Note that this significant motion will occur during the image recording, severely distorting the recorded image (Ref. 59, Fig. 2C). Figures 4(b) and 4(c) show examples of simulated free-space random-walk trajectories of a QD and an IgG molecule and show RMSDs for 10-ms and 100-ms long recordings relative to the median width of brain ECS.

The examples above illustrate that when a light source of light is moving while being imaged, motion blur changes the distribution of fluorescence in experimental images. The resulting fluorescence distribution of the image is a function of the PSF, laser beam trajectory, and the trajectory of the tracked light source in 3D. The blur is relatively small within individual lines and relatively large between different lines when scanned with unidirectional galvo-scanning. Consequently, the recorded image is similar to a vertical stack of one-pixel-tall horizontal stripes that show snapshots of the same object recorded at different times (Ref. 59, Fig. 1C).

The best solution for the problem is to record images faster. Bidirectional scanning [Fig. 4(a)], available on most of the commercial TPM, speeds up scanning, thanks to no retracing of the laser beam, but it also introduces artifacts to recorded images: intensity distributions recorded while scanning in different directions appear shifted relative to each other. This happens because of the varying speed of the laser beam, effectively resulting in images with pixels of varying widths. Only uni-directional scanning ensures a constant speed of the laser beam and hence a constant pixel width. Software for the commercial TPM typically includes a tool for correcting this artifact. However, manually corrected images or images with nonconstant pixel width, i.e., images plotted correctly based on the known time dependence of the laser beam speed, vastly complicate localization. Some gain in scanning speed can also be achieved by increasing the pixel size. Optimally, the pixel width should be as large as possible for fast scanning, but it should not exceed the SD of the PSF by much, as this will compromise localization precision.^42^ From a wide spectrum of advanced fast-TPM setups,^60^^–^^63^ random-access scanning with acousto-optical deflectors ^64^^–^^67^ is a powerful approach for speeding up single-particle tracking.

If no advanced hardware solutions are available, similar to an average user of a commercial TPM, can data analysis still locate the tracked objects on images distorted by the motion blur? In wide-field microscopy, optimal localization analysis has been developed both for stationary objects^42^ as well as for moving objects, e.g., in the case of confined diffusion.^68^ These theories, however, do not apply to scanning microscopy with a motion blur that is more severe and prohibitively complex to model. Deep learning algorithms, on the contrary, do not rely on an explicit physical theory of the blurring and can estimate diffusion coefficients of QDs in the brain ECS *in vivo* from images contaminated with severe motion blur.^59^ This is facilitated by the relative ease with which one can generate realistic training data for training deep neural networks.^59^

### 3.3 Motion Blur Due to Blood Flow

**Fig. 5:**
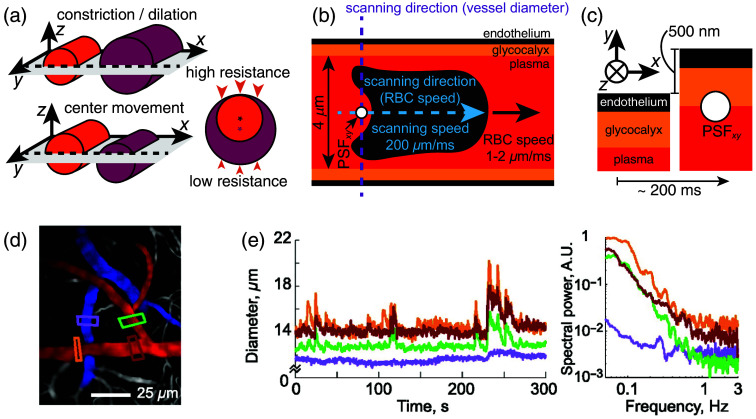
(a) Two types of vessel motion: constriction/dilation (top) and movement of the center (bottom). The cartoon on the right shows how the two can be related: a dilation causes movement of the vessel’s center (asterisks) if the stiffness of the surrounding tissue varies around the vessel. (b) The microscope’s PSF (seen in xy-plane) relative to the lumen, glycocalyx, and endothelium of a 4-μm diameter capillary. When measuring vessel diameters and RBC speed, the vessels are scanned perpendicular or parallel to the vessel’s cylinder axis, respectively. (c) Peak-to-peak amplitude of an arteriole’s diameter pulsation, ΔR≈500  nm^2^ shifts the endothelium and the glycocalyx position, introducing significant motion blur. The thickness of the glycocalyx and the endothelium, as well as the dimensions of the PSF, is as in Fig. 1a. (d) Surface brain arteries (red) and veins (blue). Rectangles show locations of vessel diameter measurements.^69^ (e) Vessel diameters (left) measured from locations depicted in panel (d) and their corresponding power spectra (right),^69^ showing $∼1/f^{2}$ dependency on top of the white-noise floor. For more examples of this dependency, see inset in (Ref. 70, Fig. 2C). Panels (d) and (e) are from (Ref. 69, Fig. 1BC), PNAS, with permission.^31^

Motion blur by diffusion is the most fundamental source of motion blur, present when imaging cell cultures, brain slices, or living brains in *in vivo* setups. Motion blur in *in vivo* imaging, however, is not limited to diffusion but also comes from the motion of blood vessels, which consists of two components [Fig. 5(a)]. First—dilation or constriction of a vessel. Second—motion of the center of a vessel, i.e., the vessel is not changing its diameter, but it moves as a whole. The two types of motion can be correlated: dilation of a vessel can potentially cause drift of the vessel’s center if nonhomogeneous surroundings interfere [Fig. 5(a)]. Changes in a vessel diameter and a vessel’s center position can be observed when following the motion of the vessel driven by the heartbeat, which creates two pressure waves.^71^^–^^74^ First propagates inside blood vessels and causes pulsations of their diameters. The second propagates through the brain tissue. As a result, the centers of the vessels move with the pulsating brain tissue. This proposed motion agrees with experimental data showing how the spectral power of heartbeat pulsations of the diameter decreases with increasing capillary order (due to the decreasing pressure drop along capillaries of increasing orders) and how the spectral power of heartbeat pulsations of the vessel centers does not depend on the capillary order.^74^ The latter happens because centers of capillaries of different orders are pulsating in synchrony with the brain tissue that contains them.^74^

Pulsations of vessel diameters and centers can cause severe motion blur. Figure 5(c) shows how a 500-nm pulsation of the diameter of a 24-μm-diameter arteriole (Ref. 2, Fig. 2E) causes both the endothelial cell and endothelial glycocalyx, coating its luminal surface, to move 500 nm—the distance exceeding their widths [Fig. 5(c)]—in just a fraction of a heartbeat. This blurring will compromise the localization of the endothelium and the glycocalyx and will not allow for correct estimation of the glycocalyx width—the most commonly used metric for characterizing the glycocalyx.^25^ If one is tracking nanoparticles close to a pulsating blood vessel, the nanoparticles’ locations will likely be affected by the motion of the vessel wall.

Heartbeat-driven pulsations are not the only source of motion blur in *in vivo* imaging. Table 1 lists five various types of motion of blood vessels that can introduce motion blur. For example, locomotion can cause a several-μm-change in a vessel diameter shown in Figs. 5(d) and 5(e). Even in the absence of locomotion or any external stimulation, blood vessel change their diameters following *spontaneous* activity. This spontaneous motion of blood vessels is responsible for correlated changes in vessel diameters, which are seen in the power spectra, P(f), of diameter time traces as $P(f)\propto f^{-2}$ noise [Fig. 5(e)]. Finally, note how some amplitudes of vessel motion are several μm or higher, exceeding the width of the diffraction-limited PSF by several-fold, which will distort the images of blood vessels if they are imaged slower than the characteristic times of their natural motion (Table 1).

**Table 1:** Factors modulating the diameter of blood vessels and causing drift of vessels with brain tissue.

Heartbeat	**Periodic** change of vessel diameter (also RBCs speed and flux) and the vessel center position driven by pressure waves generated by heartbeats and propagating through blood vessels and brain tissue. The latter is driven by the pulsation of large brain arteries.^71^^,^^75^
	**Amplitude**: 1 μm^b^ fluctuations of diameters in 60 μm pial arteries (Ref. 11, Fig. 3d). In capillaries, RBC speed increases by 28±12% in Ref. 76 and up to 15% in Ref. 77, where the effect decreased with increasing branching order of vessels.^76^ Pulsation of the vessels centers: ≤1.5 μm^c^,^78^ and ≤1 μm^c^ (average 0.35 μm),^79^ both measured in anesthetized mice.
	**Frequency**: 5 to 10 Hz in anesthetized mice (depends on anesthesia).^11^^,^^77^
Vasomotion	**Periodic** fluctuation of vessel diameter in arteries but not in veins.^80^ Vasomotion is believed to connect slow electric brain signals to changes in local blood oxygenation,^81^ maintains tissue perfusion,^82^ and drives clearance of brain tissue.^80^
	**Amplitude**: 2 to 3 μm^a^ for a 20-μm arteriole^80^; 3 μm^a^ for a 35 μm arteriole.^81^
	**Frequency**: 0.1 to 1 Hz.^69^^,^^80^^,^^81^
Breathing	**Periodic** brain movement, possibly caused by changing blood or (and) intracranial pressures.^76^^,^^83^
	**Amplitude**: ≈1 μm brain displacement along one dimension in awake rats (Ref. 83, Fig. 4B).
	**Frequency**: ≈1 to 2 Hz in awake rats^83^ and 1 to 3 Hz in awake mice.^84^^,^^85^
Locomotion	**Transient** response to locomotion, which correlates with the running speed of a mouse.^86^
	**Amplitude**: 9 μm^a^ (43%) dilation of a 20-μm penetrating arteriole of an awake mouse during natural whisking/running^87^; 5 μm^b^ (≈25%) constriction of 18-μm dural vessels of an awake mouse during locomotion (Ref. 88, Fig. 2). Movement of the vessels centers with the brain: $\approx 1\;\mu m\;s^{-1}$^b^ in mice (Ref. 86, Fig. 2); up to 30 to $40\;\mu m\;s^{-1}$^b^ in rats (Ref. 83, Fig. 2B). Drift of vessels centers with mean maximum speed up to $0.14\pm 0.1\;\mu m\;{\mathrm{ms}}^{-1}$ and the highest speed of $0.32\;\mu m\;{\mathrm{ms}}^{-1}$.^89^
	**Duration**: sec to tens of sec.^88^
Stimulation	**Transient** response, typically caused by the neurovascular coupling, to an external stimulus, e.g., whisker stimulation.
	**Amplitude**: 15 μm^b^ dilation of a 50-μm arteriole (Ref. 90, Fig. 2); 4-μm^b^ dilation of a 10-μm arteriole of an awake mouse (Ref. 69, Fig. 2A); 2.4-μm^b^ dilation of a 20-μm arteriole of an awake mouse (Ref. 87, Fig. 1); 0.1 μm^a^ dilation of a 4-μm capillary^91^; 0.22 μm^a^ dilations of single capillaries (4.4 μm average diameter).^92^
	**Duration**: sec to min.^90^^,^^92^^,^^93^

a Calculated based on the reported value, relative to the resting diameter of a vessel.

b Calculated approximately based on visual inspection of the cited figure.

c Heart beat + breathing.

### 3.4 Faster Imaging—Less Motion Blur but More Noise

**Fig. 6:**
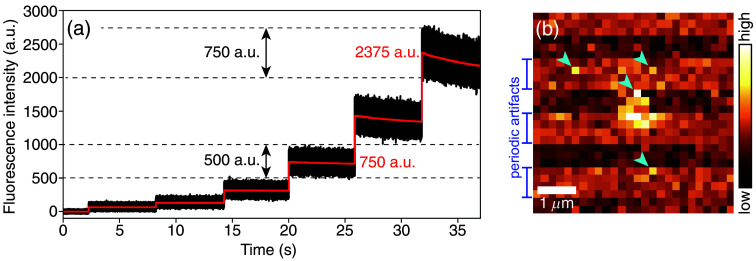
Signal and noise in two-photon microscopy. (a) Black curve: fluorescence intensity recorded in time from a single point (no scanning) inside a plastic fluorescent slide (FSK5, ThorLabs, USA) at seven increasing laser intensities, starting at zero (data from Kutuzov et al.^94^). Because data points were sampled with high inverse sampling rate of 2  μs, the data appear as black bands with indistinguishable individual values on the time-axis spanning half a minute. Red curve: block-averaged black curve (blocks of 8 ms) showing averaged fluorescence intensity from the fluorescent slide. The averaging removes the noise (as judged by eye); therefore, the red curve represents the *signal*—expected value of fluorescence intensity. Note how both the *signal* (red) as well as the height of black bands (estimates *noise*—random fluctuation about the *signal*) increase with increasing laser intensity. However, the *signal* increases faster than the *noise*: a roughly three-fold increase in signal (2375 a.u./750 a.u.) corresponds to only 1.5 increase in noise (750 a.u./500 a.u.). This simple experiment illustrates the *shot noise*—the most fundamental source of noise on optical images—and shows why we should always collect as many photons as possible: More photons mean a higher signal-to-noise ratio. Note the decay of the red curve with the highest intensity values, which is explained by the photobleaching of the dye in the plastic slide. Photobleaching and undesirable photoablation limit the maximum laser power used in a typical *in vivo* experiment. (b) Image of a quantum dot (QD, bright blob in the middle) in the brain ECS of a living anesthetized mouse, recorded with TPM (data from Kirkegaard et al.^59^), showing periodic artifacts introduced by the microscope^94^ and single-pixel-noise. Cyan arrowheads point to some of the brightest pixels in the image. Statistical analysis of TPM images, based on probability distributions of single-pixel outputs (Ref. 94, Fig. 8), can help determine whether these pixel values are consistent with a constant background around the QD in the middle. Or whether these bright pixels show, e.g., fluorescence of an out-of-focus QD.

Fast image acquisition helps to avoid, or at least minimize, motion blur, which can compromise accurate localization of the moving objects. Because TPM is slow, for many applications, an experimenter cannot afford to average recorded images in time to improve signal-to-noise ratio (SNR), which is commonly done when fast image recording is not a priority. Furthermore, it is not always possible to increase the laser power until the desired image brightness is achieved due to photobleaching or photodamage of the brain tissue. This results in noisy images. Figure 6(a) explains the *shot noise*—the most fundamental source of noise in optical microscopy. Note that shot noise is not the only noise present in images recorded with TPM.^94^

Consider an image of a QD, diffusing in the brain ECS [Fig. 6(b)].^59^ The image consists of three main components: (i) background, consisting of photonic background of the brain (autofluorescence + photons from out-of-focus QDs) and dark output of the detector—a photomultiplier tube (PMT)—when operated in complete darkness^94^; (ii) periodic bias (specific to a microscope’s setup^94^); and (iii) signal generated by photons emitted by the QD. Suppose our task is to localize the QD, which can be a relevant experiment for studying drug delivery across the blood-brain barrier. Here are several examples of questions we need to answer: are the values of four bright pixels marked by the arrowheads [Fig. 6(b)] consistent with a uniform image background and high fluorescence values are *noise*? Or do they show contributions from photons emitted by the QD we are tracking, i.e., they are the *signal*? There is no doubt that analyzing noisy images is much more complex than the analysis of well-averaged images, where the noise is almost indistinguishable on top of the smooth fluorescence intensity in the images. An optimal localization analysis that handles noisy images has been developed for WFM.^42^ It is based on modeling image formation using realistic PSF as well as knowing statistical distributions of the detector outputs, i.e., of CCD or EMCCD cameras. Similarly, one can characterize the statistical properties of PMTs used in TPM, which requires only several simple model experiments, similar to one explained in Fig. 6(a).^94^ Finally, one needs to experimentally measure, or at least theoretically estimate, the PSF and quantitatively model image distortion due to the motion blur. In some cases, this can easily become prohibitively complex, which is why fast tracking of transport processes from a noisy image is one of the biggest challenges of TPM.

## 4 Practical Considerations for Optimal Data Collection and Analysis

In this section, we provide practical suggestions to help maximize the quality of recorded images. Although not exhaustive, these recommendations are informed by the challenges and limitations of TPM discussed throughout this review. We believe the following tips can significantly enhance the reliability of routine TPM measurements and support the exploration of more demanding and innovative applications.

### 4.1 Make Null Experiments

The goal is to ensure your microscope is properly functioning.

1. Record dark output (signal recorded with zero photon input, i.e., in complete darkness) of the PMTs to ensure stable output (no PMT malfunctioning), no parasitic signals or undesired artifacts (Ref. 94, Fig. 1).
2. Record fluorescence output from a fluorescent slide [Fig. 6(a)] or a solution of a fluorophore to ensure stability of the laser power as well as the PMTs’ output at nonzero photon input.
3. Record images of small (e.g., 40 to 100 nm beads are commercially available) fluorescent beads to characterize the microscope’s PSF and compare it with the expected PSF for the diffraction-limited imaging (Fig. 1). Distortions of the measured PSF can indicate, e.g., a misaligned laser in the optical path of the microscope.

### 4.2 Select Optimal Image Acquisition Parameters

The goal is to record data optimally to facilitate and simplify data analysis.

1. If you can increase the laser power without risking excessive photobleaching or photodamage, do it. This will ensure the highest signal-to-noise ratio on recorded images [Fig. 6(a)].
2. Avoid applying too high voltages to your PMTs (HV, high voltage). Increasing the voltage on PMTs increases the number of false-positives, i.e., seeing a bright pixel in the absence of photons (Ref. 94, Fig. 5).
3. For localization tasks, especially for tracking small structures with dimensions comparable with the wavelength of the excitation laser light (e.g., nanoparticles, endothelial glycocalyx), pixel size should not exceed the SD of the PSF by much, as this will compromise localization precision.^42^
4. Select the image sampling speed based on (i) the temporal scale of the studied process, e.g., based on the RMSD calculations in Fig. 4 if imaging diffusion, and on (ii) potential motion blur artifacts due to, e.g., motion of blood vessels (Table 1).

### 4.3 Calibrate Your PMTs

The goal is to (i) facilitate interpretation of the recorded fluorescence intensity, expressed in *photons* instead of *arbitrary units*^94^; (ii) allow comparisons of fluorescence intensities, now measured in *photons*, recorded with different acquisition parameters (voltage on PMTs); and (iii) perform advanced data analysis, e.g., super-localization. To calibrate your PMTs, follow Ref. 94 (Sec. V, Fig. 7) and Ref. 94 (Secs. VI, VII) for more advanced tasks.

### 4.4 Choose Optimal Data Analysis

The goal is to obtain the most accurate and precise measurements.

1. For localization on recorded images, use maximum-likelihood estimators (MLE), developed based on the physical description of the image formation and statistical properties of the photon detectors (EMCCD cameras, PMTs) in the images.^42^^,^^94^
2. If the fluorescence distributions on recorded images are too complex (e.g., due to severe motion blur) to be explained with explicit physical models, deep neural networks can be used.^59^
3. When determining transport mechanisms and estimating diffusion coefficients from single-particle trajectories, choose the covariance-based estimator^46^^,^^95^ over a more commonly used standard MSD-based analysis.

## 5 Summary

TPM remains the gold standard for high-resolution imaging in scattering media similar to the brain. Studying transport *in vivo* at the microscale—at the level of single cells—is an ultimate challenge for TPM. First, because TPM employing red-light excitation has lower spatial resolution compared with, e.g., confocal microscopy, which puts a limit on the distances at which we can resolve and analyze the transport. This is a problem because key biological structures affecting the transport, namely the glycocalyx, vascular endothelium, and single channels of the brain ECS, are all smaller than the wavelength of light used in TPM; hence, the spatial resolution of TPM is not good enough to resolve the transport within these structures. On the contrary, images recorded with TPM are not distorted by the light scattering of the brain compared with images recorded with WFM (IOI method). Second, because TPM is slow because of the pixel-by-pixel image acquisition (scanning), the tracked objects can move considerably during the exposure, sometimes leading to severe motion blur. To illustrate this, we explained how the natural transport of molecules in the brain driven by diffusion introduces motion blur, which will further be largely exacerbated by the natural motion of blood vessels in the brain, e.g., by the heart-driven vessel wall pulsations and vasomotion. Finally, we addressed strategies for robust statistical analysis of noisy images, typically recorded with low-photon budgets *in vivo*. Together, these methodological aspects of the spatial and temporal resolution of TPM provide guidelines for planning an experiment and interpreting its results with the goal of maximizing the effectiveness of TPM in probing transport dynamics in the living brain.

## Data Availability

The authors have not used any code or collected any data in this paper.
